# Daytime Sleep Enhances Consolidation of the Spatial but Not Motoric Representation of Motor Sequence Memory

**DOI:** 10.1371/journal.pone.0052805

**Published:** 2013-01-02

**Authors:** Geneviève Albouy, Stuart Fogel, Hugo Pottiez, Vo An Nguyen, Laura Ray, Ovidiu Lungu, Julie Carrier, Edwin Robertson, Julien Doyon

**Affiliations:** 1 Functional Neuroimaging Unit, C.R.I.U.G.M., Montreal, Canada; 2 Psychology Department, University of Montreal, Montreal, Canada; 3 Psychiatry Department, University of Montreal, Montreal, Canada; 4 Department of Research, Donald Berman Maimonides Geriatric Center, Montreal, Canada; 5 Centre of Advanced Research in Sleep Medicine, Hôpital du Sacré-Coeur de Montreal, Montreal, Canada; 6 Harvard Center for Noninvasive Brain Stimulation, Harvard Medical School and Beth Israel Deaconess Medical Center, Neurology Department, Boston, Massachusetts, United States of America; Queensland Brain Institute, Australia

## Abstract

Motor sequence learning is known to rely on more than a single process. As the skill develops with practice, two different representations of the sequence are formed: a goal representation built under spatial allocentric coordinates and a movement representation mediated through egocentric motor coordinates. This study aimed to explore the influence of daytime sleep (nap) on consolidation of these two representations. Through the manipulation of an explicit finger sequence learning task and a transfer protocol, we show that both allocentric (spatial) and egocentric (motor) representations of the sequence can be isolated after initial training. Our results also demonstrate that nap favors the emergence of offline gains in performance for the allocentric, but not the egocentric representation, even after accounting for fatigue effects. Furthermore, sleep-dependent gains in performance observed for the allocentric representation are correlated with spindle density during non-rapid eye movement (NREM) sleep of the post-training nap. In contrast, performance on the egocentric representation is only maintained, but not improved, regardless of the sleep/wake condition. These results suggest that motor sequence memory acquisition and consolidation involve distinct mechanisms that rely on sleep (and specifically, spindle) or simple passage of time, depending respectively on whether the sequence is performed under allocentric or egocentric coordinates.

## Introduction

How did Mozart manage to play his sonatas backwards on a piano? Although the behavioral mechanisms involved in such a virtuosos performance remain largely unknown, part of the answer may reside in research conducted in the last two decades aiming at understanding the different levels of representation through which new motor sequences can be learned [1]. Indeed, motor sequence learning has been shown to encompass two independent processes named “spatial” and “motor” [2]–[4]. For example, a pianist performs a series of sequential finger movements (motor representation) to play notes in order to achieve a particular piece of music (i.e., goal of the movement or spatial representation). Yet, these two components of learning seem to progress with different time courses: While the spatial process is believed to be elicited rapidly in the early learning phase under high control and attentional demands, the motor process is thought to be acquired more slowly under automatic modes [2]–[4]. In line with this model, several behavioral studies have used experimental protocols designed to look at the transfer of motor sequence knowledge from one coordinate space to another (e.g., from one hand to the other, or from one keyboard configuration to another) to determine the nature of the representations underlying such processes (for a review, see [1]). For example, a pianist would be asked to play a known sonata backwards on a piano. In this new configuration, the same motor movements are no longer associated with the same sequences of note (not the same melody); it would then be possible to test both motor (movements) and spatial (melody) representations of the learned sonata. Accordingly, the spatial representation of a motor sequence, also referred to as the perceptual [5] or abstract representation [6]–[8] by other investigators, would represent the goal of the series of movements that need to be executed under allocentric [9] or extrinsic [10] coordinates, i.e. in an external frame of reference. Such an effector-independent representation of the sequence [11]–[13] has been thought to rely mainly on activity of the prefrontal and parietal cortices [2]–[4], [6]. By contrast, the motor representation [14] would constitute a more intrinsic, movement-based skill realized under egocentric coordinates [9], in an internal frame of reference. This effector-dependent representation of the sequence [6], [8], [11]–[13] has been found to recruit motor-related structures [2], [3], [6], [8], [15], [16].

Motor sequence memory consolidation can be characterized by a spontaneous improvement in performance observed between practice sessions, without any further training [17]–[19]. In most cases, such performance gains are observed only if this interval contains a period of nocturnal [20]–[24] or diurnal sleep [24]–[26], but not with the simple passage of time. These findings are consistent across studies with respect to explicit motor sequence learning, but one should note, however, that the role of sleep in consolidation of implicit motor sequence memory appears less crucial [21], [27], [28]. Controversial results are also observed regarding the implication of the different sleep stages in the consolidation process: while some studies show that rapid eye movement (REM) sleep appears to facilitate motor sequence memory consolidation [9], [29], [30], there is increasing evidence that non-REM (NREM) sleep does play a crucial role in this process [10], [25], [26], [31]–[35]. In particular, sleep spindles, which are brief electrophysiological events of NREM sleep predominantly observed during stage 2 sleep and believed to reflect mechanisms of synaptic plasticity and long-term potentiation [36], have previously been associated with better consolidation of a novel motor sequence [26], [32]–[35].

While there is now a great deal of accumulated evidence regarding the role of sleep in the consolidation of motor sequence learning, the contribution of this physiological state in the consolidation of either the allocentric or egocentric representation of a newly acquired sequence of movements has only recently been studied. Behavioral studies have demonstrated that a night of sleep, and NREM stage 2 sleep in particular, aids the expression of the allocentric representation of a motor sequence [10], while the simple passage of time appears to be sufficient to facilitate the expression of its egocentric representation [37]. Yet the latter studies do not offer direct insights into the possible effects of sleep on consolidation of such representations. Interestingly, this issue has been addressed more directly in an elegant study reported by Cohen and colleagues [9] who found a clear double dissociation in consolidation processes between the two different representations, hence suggesting that distinct systems enhance the different aspects of a memory trace: While the spatial representation of the sequence was consolidated following a period of nocturnal sleep, the motor representation was consolidated after an equivalent wake period [9]. While compelling, the latter study still left some unanswered questions. First, the authors used an implicit version of the serial reaction time (SRT) task, the consolidation of which is believed to occur over wakefulness rather than over sleep [21]. Second, the transfer effect ensuring that subjects learned the two representations of the sequence after initial training was not tested, and thus possible confounding factors such as the time of testing during the day (i.e., circadian confound) and fatigue effects known to overestimate offline gains in performance [38], [39] were not controlled for. Finally, although Cohen et al. [9] reported that REM sleep was correlated with the consolidation of the allocentric representation of the sequence, it is still possible that NREM sleep [10], and spindles in particular, may be involved in this process.

**Figure 1:**
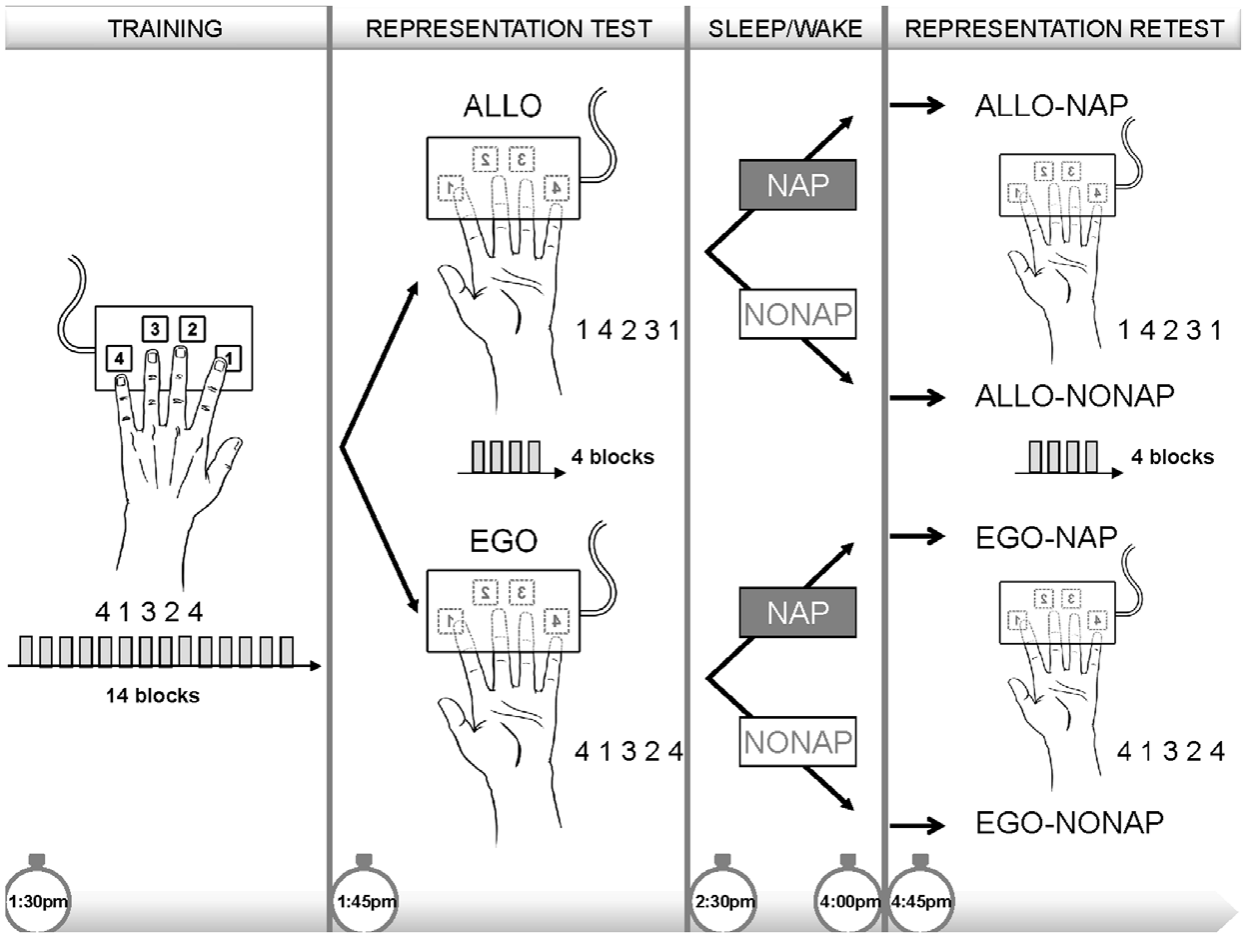
Task and experimental protocol. Training panel: All the subjects were trained on the FTT with the usual set-up (hand on the keypad). Representation test panel: After initial training, switching the keypad and hand coordinates by turning it upside down, allowed to distinguish between two types of representation of the sequence: the spatial allocentric (ALLO, same spatial sequence but different finger movements) and motor egocentric (EGO, same finger movements but different spatial sequence) representations. Representation retest panel: After a 90-minute nap (NAP) or a wake period (NONAP), all subjects were retested on the representation they were trained on.

In the present study, we thus used an explicit sequential finger tapping task (FTT, Figure 1, Training session, sequence - 4 1 3 2 4 -) to characterize the effect of daytime sleep (nap) *vs.* wakefulness on the consolidation of both allocentric and egocentric representations of the sequence. The existence of these two representations after initial learning was measured using a “transfer” protocol in which all subjects were tested on their ability to produce the motor or spatial sequence with the same hand, but with the keypad turned upside down (Figure 1, Representation Test session). By reversing the keypad, the same finger movements were no longer associated with the identical spatial sequence and vice versa. Accordingly, such a manipulation generated two different sequence representations: an egocentric (EGO) representation that probed movement-based learning (i.e., same motor movements - 4 1 3 2 4 - that produced a different spatial sequence) and an allocentric (ALLO) representation that probed spatial-based learning (i.e., same spatial sequence - 1 4 2 3 1 -, which required subjects to produce a different sequence of movements, see Figure 1, Representation Test session). After this test session, participants were divided into two groups according to whether they were allowed to take a 90-minute nap (NAP) or were asked to stay in quiet wakefulness (NONAP). Subjects in the four experimental groups (ALLO-NAP, ALLO-NONAP, EGO-NAP and EGO-NONAP) were then retested 45 minutes after the NAP/NONAP period on the same representation they were trained on, hence controlling better for the time of day difference between sessions (Figure 1, Representation Retest session). Changes in performance between test and retest sessions, observed after daytime sleep or wakefulness, were taken as an indicator of offline consolidation for the two representations of motor sequence learning. Finally, the possible impact of fatigue on this indicator was also controlled for.

We hypothesized that: (1) both allocentric and egocentric representations of the sequence would be segregated after initial training and that (2) daytime sleep would favor the consolidation of the allocentric, but not the egocentric representation of the sequence. Testing for the contribution of REM or NREM sleep, and specifically NREM sleep spindles, in the consolidation of the allocentric representation of the sequence was more exploratory in nature.

## Materials and Methods

### Ethics Statement

All the participants gave their written informed consent to take part to the study which was approved by the Research ethics board of the RNQ (Regroupement en Neuroimagerie du Québec). They were paid for their participation to the study.

### Population

Forty-eight young (mean age: 24±3.8 years, 19 females) right-handed [40] healthy volunteers were recruited by local advertisements to participate in this study. They had no history of medical, neurological or psychiatric disease. None of the subjects were taking medication at the time of testing. Also, none of them had ever played a musical instrument nor was trained as a typist. The quality of their sleep was normal as assessed by the Pittsburgh Sleep Quality Index questionnaire [41] and the St. Mary Hospital questionnaire [42].

### Motor Sequence Learning Task

The subjects’ performance in motor sequence learning was assessed over 3 separate sessions referred to as the training, the representation test and the representation retest sessions. On each occasion, they were asked to practice a sequential finger tapping task coded in Cogent2000 (http://www.vislab.ucl.ac.uk/cogent.php) and implemented in MATLAB (Mathworks Inc., Sherbom, MA). The task required that subjects tap on a keyboard, with their (left) non-dominant hand, a five-element finger sequence as rapidly as possible while making as few errors as possible. The sequence to perform (4 1 3 2 4, where 1 corresponds to the index and 4 to the little finger, Figure 1) was explicitly thought to the participants prior to training and constantly displayed on a screen during practice. This task was performed in 14 successive practice blocks during the training session and 4 successive practice blocks during the representation test and retest sessions, each practice block separated by 15-second rest periods (Figure 1). The task was coded to record the number of key presses within a block (maximum 60 key presses). After 60 key presses, the “practice block” automatically changed to a “rest block” where subjects were simply required to look at a fixation cross. Such a procedure permitted the control of the number of movements executed in a block. Yet, the duration of the practice blocks progressively decreased with learning as subjects became faster on performing the 60 key presses (i.e., 12 possible sequences).

Motor skill performance was measured in terms of speed (block duration to perform the 60 key presses) and accuracy (number of errors by block). Supplemental fine-grained analyses on speed (i.e., 3 averaged measures of speed per block representing the time to perform the first 20 (1 to 20), second 20 (21 to 40) and third 20 (41 to 60) key presses) were also performed to assess possible within-block fatigue effects [38].

### Experimental Procedure

All subjects performed the same training session, which consisted of 14 blocks with the trained sequence - 4 1 3 2 4 -, in the early afternoon (around 1∶30 p.m.), using the usual testing setup, i.e, the left hand positioned on the keypad (Figure 1, left panel, Training). After training, subjects were then assigned to one of two groups depending on whether they were going to be tested on the allocentric (ALLO) or egocentric (EGO) representation of that sequence. Performance on the allocentric and egocentric representations of the sequence were tested during the representation test session that comprised 4 blocks of practice during which the keyboard and the subject’s hand were turned upside down (see Figure 1, middle panel, Representation Test). The allocentric representation was thus assessed by changing the specific pattern of finger movements that subjects needed to perform, while preserving the spatial representation of that sequence (from sequence - 4 1 3 2 4 - to its mirror configuration - 1 4 2 3 1 -). By contrast, the egocentric representation of the sequence was assessed by changing the locations of the movement responses, hence preserving the specific pattern of sequential finger movements learned during training (i.e., sequence - 4 1 3 2 4 -). At the end of the representation test session (around 2∶00 p.m.), subjects were again pseudo-randomly divided, in an alternating fashion, into two further groups according to whether they were allowed to take a 90-minute nap (NAP) or to stay in quiet wakefulness for the same amount of time (NONAP). Each nap period was monitored using standard polysomnographic recording materials and procedures (see details in *Polysomnographic data acquisition and analyses* section). In the NONAP groups, subjects were required to rest with their eyes open and were allowed to read magazines while lying on a bed under dim light condition during the 90-minute waking period, which remained under the constant supervision of the experimenters to ensure that subjects did not fall asleep. Subjects in the four groups (ALLO-NAP, ALLO-NONAP, EGO-NAP and EGO-NONAP) were then retested using four additional blocks of practice on the sequence representation in which they were trained. This retest session was administered 45 minutes after the end of the NAP/NONAP periods (around 4∶45 p.m., Figure 1, right panel, Representation Retest) to ensure dissipation of sleep inertia. A psychomotor vigilance task (PVT, [43]) was also administered before the retest session in each group in order to compare the level of vigilance between sleep and wake conditions.

### Polysomnographic Data Acquisition and Analyses

Nap periods were recorded with a digital ambulatory sleep recorder (Vitaport-3 System; TEMEC Instruments, Kerkrade, The Netherlands) and were digitized at a sampling rate of 512 Hz using commercial software (Colombus). Standard electroencephalographic (EEG) recordings were made from Fz, C3, Cz, C4, Pz, Oz, A1 and A2, with A2 used as the recording reference and A1 as a supplemental individual EEG channel. An electrode placed on the middle of the forehead was used as the recording ground. Bipolar vertical and horizontal eye movements (electrooculogram: EOG) were recorded from electrodes placed above and below the right eye and on the outer canthus of both eyes, respectively. EEG and EOG data were recorded with a 0.1 Hz low cutoff and a 30 Hz high cutoff. Bipolar submental electromyogram (EMG) recordings were made from the chin, filtered from 10 to 200 Hz to record muscle tone and movements. Electrical noise was filtered using a 60 Hz notch.

Polysomnographic data of the diurnal sleep recordings were visually scored, with 30-s epochs, by a trained sleep technician (author LR, http://www.sleep-well.ca/) according to standard criteria [44] using the fMRI Artefact rejection and Sleep Scoring (FASST) Toolbox (http://www.montefiore.ulg.ac.be/~phillips/FASST.html - University of Liege - [45]). To easily visualize the relevant features of sleep and wakefulness, EEG was re-referenced to an average of A1 and A2 displayed from 0.5 to 30 Hz, EOG below 10 Hz and EMG above 10 Hz using software filters.

Spindle detection was carried out after down-sampling the EEG data to 150 Hz. The detection was performed on Fz, Cz and Pz derivations, referenced to the average of both mastoids (A1 and A2). The signal was filtered from 0.5 to 30 Hz, in which frequencies from 11 to 17 Hz were extracted from movement-free NREM sleep epochs (sleep stages 2, 3 and 4). The latter detection method, developed in our laboratory (author SF) with Vision Analyzer Software (Brain Products, http://www.brainproducts.com), uses a complex demodulation transformation of the EEG signals in the frequency band of interest. Then, each data point was transformed into a z-score using the mean and the standard deviation calculated from a 60 second sliding window. Events (spindle onsets, peaks and offsets) were then detected on the transformed signal with a z-score threshold of z = 2.33, equivalent to the 99^th^ percentile (*i.e.,* p = 0.01, one-tailed). This automatic detection algorithm was highly supervised by a trained sleep technician (author LR) for each step of the processing, and was finally verified visually for each subject. This method allows to extract, for each subject and at each derivation of interest (Fz, Cz and Pz), the total number of spindles and the average spindle size (area in standardized µV^2^*s). Spindle density was computed as the total number of spindles per percentage of time passed in NREM relative to the total recording time (Number of spindles/(NREM duration*100/Total Recording Time duration)). This method has been shown to be reliable as compared to expert visual scoring having a sensitivity of 85%, specificity of 90% and false positive rate of 10% [46].

## Results

### Subjects

Two subjects were discarded from the analyses: one from the ALLO-NAP group because he slept less than 10 minutes during the 90-minute nap period, and one in the EGO-NAP group because he practiced an incorrect sequence during the representation test session. Consequently, 46 subjects were included in the analyses: 13 subjects in the ALLO-NAP group (mean age: 23.3±3.9 years, 5 females), 11 in the ALLO-NONAP group (mean age: 22.3±3.7 years, 4 females), 11 in the EGO-NAP group (mean age: 26.2±2.8 years, 7 females) and 11 in the EGO-NONAP group (mean age: 23±3.1 years, 3 females).

### Sleep Duration and Quality

#### Nocturnal sleep prior to the experiment

The duration and quality of each subject’s sleep in the month preceding the experiment was assessed with the Pittsburgh Sleep Quality Index questionnaire (PSQI, [41]). The four groups did not differ in terms of the estimated average sleep duration (ALLO-NAP, 7 h 28 min ±1 h 07 min; ALLO-NONAP, 7 h 30 min ±1 h 01 min; EGO-NAP, 7 h 58 min ±1 h 07 min; EGO-NONAP, 8 h 08 min ±1 h 06 min; unpaired t tests between groups, all t-statistics>−1.43, all p-values >0.16), nor in terms of their median PSQI scores (ALLO-NAP: 5; ALLO-NONAP: 4; EGO-NAP: 3; EGO-NONAP: 3; unpaired t tests between groups, all t-statistics <1.83, all p-values >0.08).

Similarly, sleep duration during the night preceding the training session, subjectively assessed using the St. Mary’s Hospital Sleep questionnaire [42], did not differ between groups (ALLO-NAP, 7 h 19 min ±34 min; ALLO-NONAP, 7 h 38 min ±1 h 08 min; EGO-NAP, 7 h 23 min ±26 min; EGO-NONAP, 7 h 34 min ±1 h 21 min; unpaired *t* tests between groups, all −0.85< t-statistics <0.65, all p-values >0.40). In addition, subjects’ sleep quality assessed through the same questionnaire (from very poor (1) to good (5)) did not differ between groups during the night preceding the training session (ALLO-NAP, 4; ALLO-NONAP, 4; EGO-NAP, 4; EGO-NONAP, 4; unpaired *t* tests between groups, all −1.16< t-statistics <1.10, all p-values >0.25). Altogether, these results show that the different groups were well matched as they had similar sleep habits during the month and the night prior to the beginning of the experimental sessions.

#### Experimental nap

Experimental daytime sleep recordings were scored according to standard criteria [44]. Unpaired t-tests revealed no difference in sleep architecture between the ALLO-NAP and EGO-NAP groups (i.e. sleeping period; total sleep time; stages 1, 2 and REM latencies; time in wake, time in stages 1, 2, 3, 4 and REM; movement time; sleep efficiency; number of arousals; number of arousals per hour; mean arousal duration) during the experimental daytime nap (Table 1). Also, further analyses did not reveal any differences in spindle density or size (area) between the ALLO-NAP and EGO-NAP groups (Table 1).

**Table 1:** Daytime sleep and spindle characteristics.

	ALLONAP	EGONAP	t(22)	p
**Daytime Sleep Characteristics**				
Total Recording Time	1 h 45 min 0 s	1 h 43 min 12 s	0.64	0.52
Sleeping Period	1 h 12 min 36 s	1 h 17 min 24 s	−0.78	0.43
Total Sleep Time	0 h 57 min 36 s	1 h 09 min 36 s	−1.72	0.09
Stage 1 Latency	0 h 21 min 0 s	0 h 15 min 36 s	0.16	0.25
Stage 2 Latency	0 h 25 min 12 s	0 h 18 min 36 s	1.60	0.20
REM Latency	0 h 44 min 24 s	0 h 55 min 12 s	−0.65	0.52
Time Awake	0 h 33 min 36 s	0 h 21 min 36 s	1.77	0.09
Time in Stage 1	0 h 6 min 36 s	0 h 4 min 48 s	1.06	0.29
Time in Stage 2	0 h 23 min 24 s	0 h 21 min 0 s	0.49	0.62
Time in Stage 3	0 h 5 min 24 s	0 h 6 min 36 s	−0.64	0.52
Time in Stage 4	0 h 22 min 48 s	0 h 28 min 12 s	0.83	0.41
Time in REM	0 h 5 min 24 s	0 h 12 min 36 s	−1.72	0.09
Movement Time	0 h 0 min 13 s	0 h 0 min 19 s	−0.50	0.61
Sleep Efficiency	55.19%	67.96%	−1.79	0.08
Number of Arousals	27.23	25.45	0.34	0.73
Number ofArousals/hour	0.50	0.38	1.15	0.25
Mean ArousalsDuration	0 h 0 min 8 s	0 h 0 min 8 s	−0.18	0.85
**Spindle Characteristics**				
***Fz Spindles***				
Size (Area)	1361.03¥	1369.40¥	−0.13	0.89
Density	7.93•	7.87•	0.16	0.87
***Cz Spindles***				
Size (Area)	1397.64¥	1376.06¥	−0.34	0.73
Density	7.79•	8.07•	−0.87	0.39
***Pz Spindles***				
Size (Area)	1407.15¥	1379.17¥	0.43	0.66
Density	7.78•	8.04•	−0.76	0.45

Experimental daytime sleep recordings were scored according to standard criteria [44]. Spindle detection was performed with a semi-automatic procedure (see methods). Unpaired t-tests were carried out to compare the sleep architecture between groups. REM: Rapid Eye Movement.

¥ Size (Area) is presented in standardized µV^2^*s.

• Spindle density is presented in number of spindles per percentage of time passed in NREM relative to the total recording time (see methods).

#### Psychomotor Vigilance Task (PVT)

The PVT was administrated right before the retest session, which took place 45 minutes after the nap/nonap periods, in order to control for possible fluctuations of vigilance between these two conditions. Unpaired t-tests showed that performance on the vigilance task did not differ between the sleep and wake conditions (Mean reaction time: NAP, 332.26±74.23 ms; NONAP, 303.49±25.71 ms; unpaired t tests, NAP vs. NONAP, t_(44) = _1.72, p = 0.09).

### Behavioral Results

Training consisted of 14 blocks of practice of the trained sequence. Knowledge of the learned sequence representations (ALLO or EGO) was tested by changing the task configuration (i.e., by reverting both the keyboard and subject’s hand) before the nap/nonap opportunity. This testing session called the “Representation test” session comprised four blocks of practice. Subjects of the four groups (ALLO-NAP, ALLO-NONAP, EGO-NAP and EGO-NONAP) were then retested on the representation they were trained on during a “Representation retest” session, which was also composed of four blocks of practice that were administered after the nap/nonap period (Figure 1).

#### Performance speed during training session

**Figure 2:**
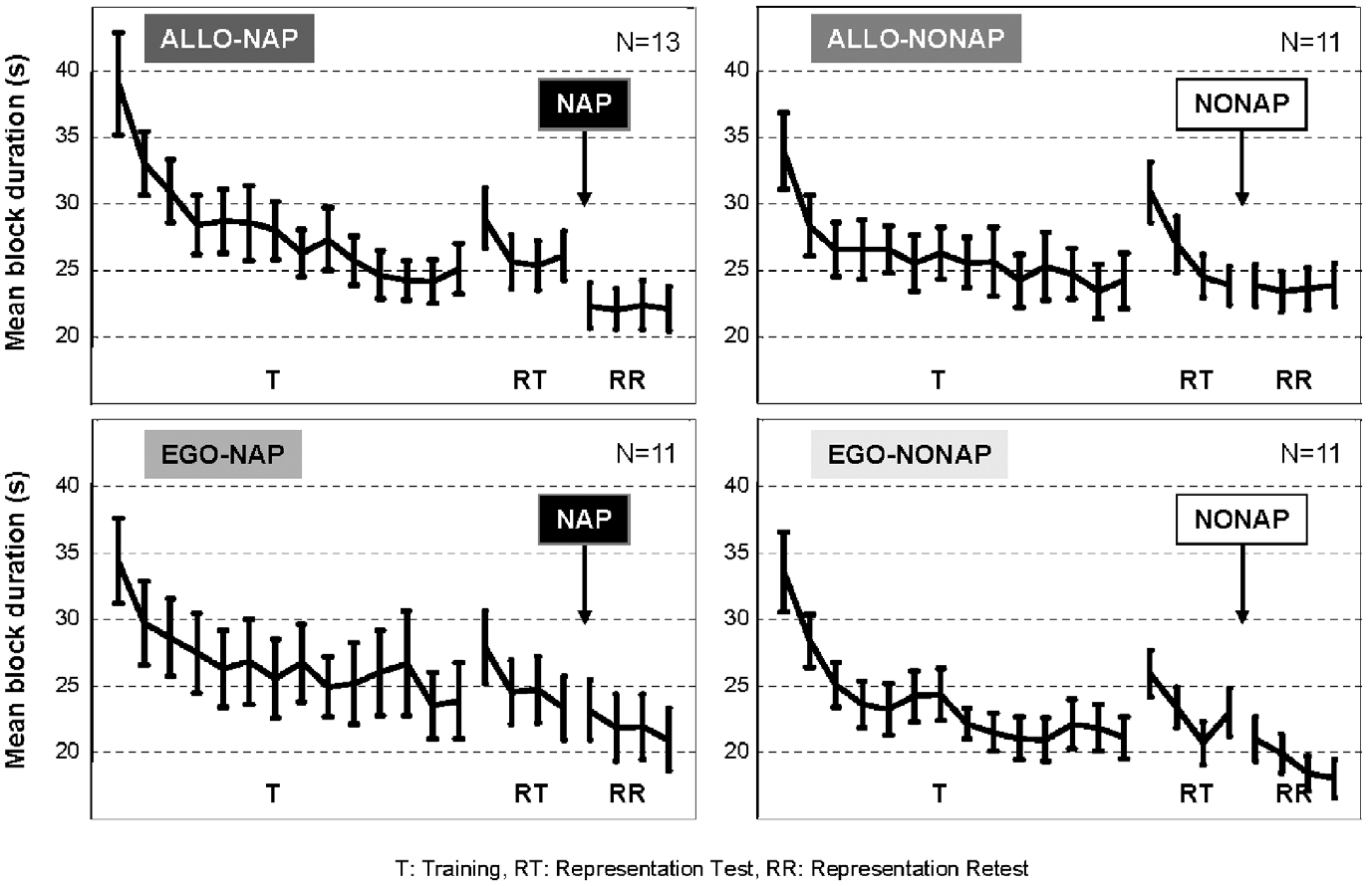
Behavioral results. Bars represent SEM. Mean block duration (s) during training (T), Representation Test (RT) and Representation Retest (RR) sessions for the ALLO-NAP, ALLO-NONAP, EGO-NAP and EGO-NONAP groups.

An ANOVA conducted on speed of performance (i.e., block duration), with the 14 blocks of practice as the within-subjects factor and group (ALLO-NAP, ALLO-NONAP, EGO-NAP, EGO-NONAP) as the between-subjects factor, yielded a significant main effect of block (F(13,546) = 33.10, p<0.0001), whereby block duration decreased with practice in all four groups. By contrast, there were no significant effect of group (F(3,42) = 0.71, p = 0.54), nor any significant block by group interaction (F(39,546) = 0.89, p = 0.65), suggesting that subjects in the four groups improved similarly on the learning task during training (Figure 2).

#### Performance speed during representation test session

An ANOVA carried out on performance speed, with blocks of practice (4 blocks) as the within-subjects factor and representation (ALLO vs. EGO) as the between-subjects factor, revealed a significant main effect of block (F(3,126) = 22.15, p<0.0001), block duration decreasing with practice for the two representations of the sequence. Importantly, there were no significant representation effect (F(1,44) = 1.49, p = 0.22), nor any significant block by representation interaction (F(3,192) = 0.19, p = 0.90), indicating that subjects performed at the same level, irrespective of the representation (spatial or motor) they were tested on (Figure 2). Furthermore, the same ANOVA performed with group as the between-subjects factor confirmed that the significant main effect of block (F(3,126) = 22.15, p<0.0001) did not differ between groups, as no significant group effect (F(3,42) = 0.62, p = 0.60), nor any significant block by group interaction (F(9,126) = 1.37, p = 0.20) were observed. These results show that the level of difficulty of the task did not differ between allocentric and egocentric conditions before the nap/nonap periods.

#### Performance speed transfer between the training and the representation test sessions

The transfer in sequence knowledge was tested before the nap/nonap period with a two-way ANOVA with the averaged performance of the first four blocks of training and the four blocks of the representation test session as the within-subject factor (session), as well as the type of representation (ALLO vs. EGO) as the between-subjects factor. This ANOVA revealed a significant main effect of session (F(1,44) = 43.19, p<0.0001), performance improving from the training to the representation test session, but no significant representation effect (F(1,44) = 1.14, p = 0.28), nor any significant representation by session interaction (F(1,44) = 0.01, p = 0.91). The same ANOVA performed with group as the between-subjects factor confirmed that the significant session effect (F(1,42) = 44.02, p<0.0001) did not differ between groups as no significant group effect (F(3,42) = 0.67, p = 0.57), nor any significant session by group interaction (F(3,42) = 1.57, p = 0.21) were observed.

**Figure 3:**
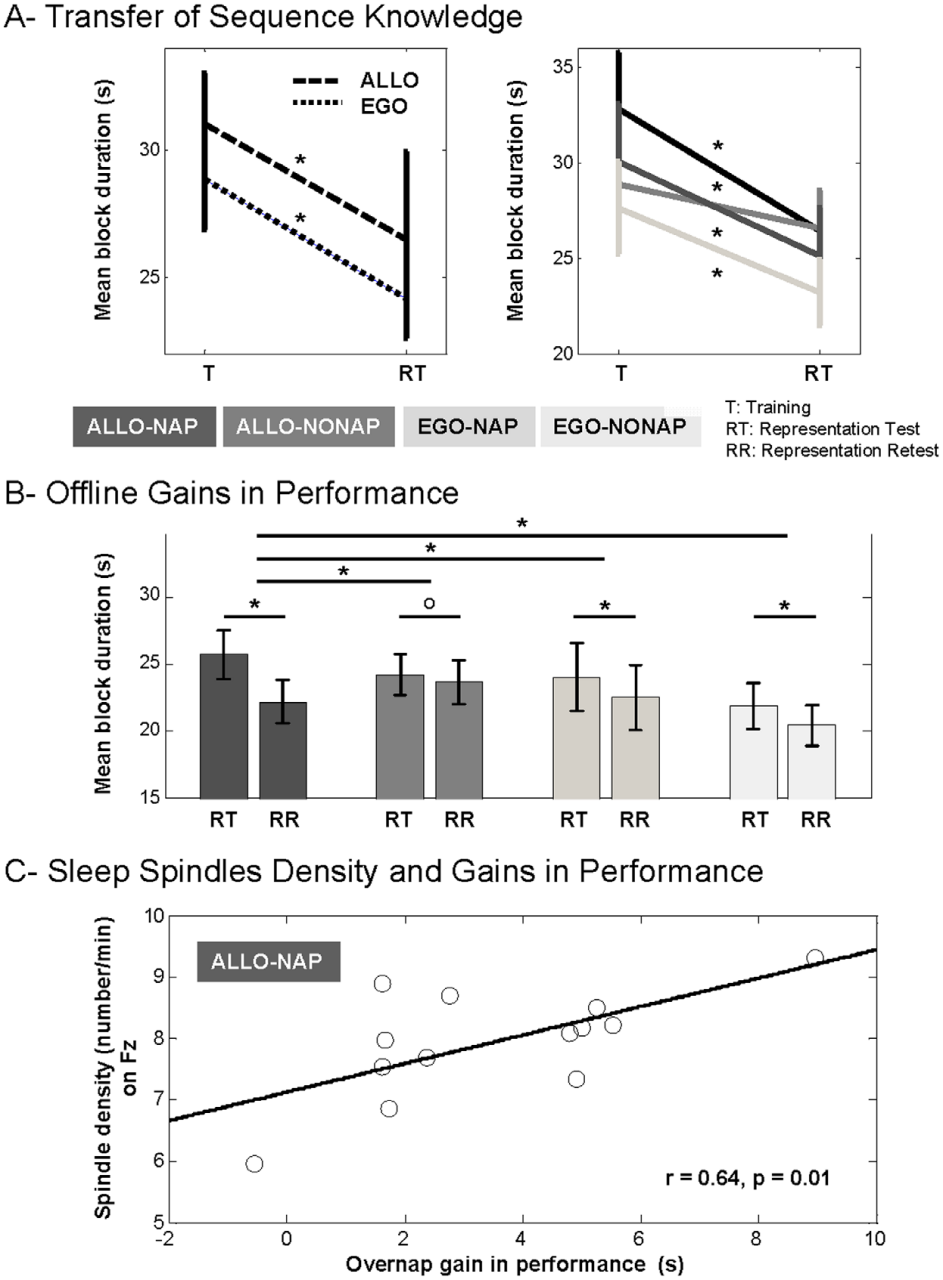
Transfer, offline changes in performance and correlation between spindle density and over-nap gains in performance for the allocentric representation. Bars represent SEM. (*) p<0.05, (o) p>0.05. A- Left panel: The transfer in sequence knowledge is illustrated by faster performance on the representation test session (with reverted keyboard) as compared to the initial blocks of training that did not differ between representations, hence suggesting the existence of 2 distinct (spatial and motor) representations of the sequence. Right panel: The effect of transfer did not differ between groups. B- Offline gains in performance are sleep-dependent for the ALLO representation whereas emerge irrespective of the sleep/wake condition for the EGO representation. When controlling for fatigue effect (data not shown), only the ALLO-NAP group showed significant offline gains in performance. C- Scatter plot showing significant correlation between spindle density (number of spindles per minute of NREM sleep relative to the total recording time) and over-nap gains in performance (s) in the ALLO-NAP group. Each data point represents a single subject of the ALLO-NAP group. Note that the correlation between spindle density and over-nap gains in performance remains significant (r = 0.62, p = 0.02) even after controlling for fatigue effects (data not shown).

Altogether, results on representation test session and transfer of sequence knowledge suggest that subjects experienced significant transfer of sequence knowledge in the new task configuration, which was independent of whether the representation of the sequence was spatial or motor in nature (Figure 3A, left panel) as performance did not differ between groups and representations during the representation test session. This also suggests that both representations might have been extracted from the initial learning in each experimental group during the representation test session (Figure 3A, right panel).

#### Between-session gains in performance speed

Between-session effects were computed comparing the average performance of the last two blocks of the representation test session against the first two blocks of the representation retest session in order to assess offline improvement after the sleep or wake period. This ANOVA revealed a significant main effect of session (F(1,42) = 27.12, p<0.0001), no significant group effect (F(3,42) = 0.51, p = 0.67), but a significant group by session interaction (F(3,42) = 3.67, p = 0.01). Within-group analyses showed a significant effect of session in the ALLO-NAP group (gain of 3517.88±691.89 (SEM) ms, F(1,42) = 30.94, p<0.0001), which was not observed in the ALLO-NONAP group (gain of 574.72±522.58 ms, F(1,42) = 0.69, p = 0.40). By contrast, a significant effect of session was observed in both EGO-NAP (gain of 1512.68±625.19 ms, F(1,42) = 4.84, p = 0.03) and EGO-NONAP groups (gain of 1416.91±801.21 ms, F(1,42) = 4.24, p = 0.04). Planned comparisons, corrected for multiple comparisons using the Fisher’s Least Significant Difference (LSD), were then performed to compare the session effects between groups. These analyses indicated that the offline gains in performance observed in the ALLO-NAP group were significantly larger than in any other group (ALLO-NAP *vs.* ALLO-NONAP, p = 0.002; ALLO-NAP *vs.* EGO-NAP, p = 0.03; ALLO-NONAP *vs.* EGO-NONAP, p = 0.02). In contrast, the offline gains did not differ between any of the other groups (ALLO-NONAP *vs.* EGO-NAP, p = 0.34; ALLO-NONAP *vs.* EGO-NONAP, p = 0.39; EGO-NAP *vs.* EGO-NONAP, p = 0.92). Together, these results demonstrate that the emergence of offline gains in performance was sleep-dependent for the allocentric representation of the sequence, but appeared irrespectively of the sleep/wake condition for the egocentric representation (Figure 3B).

**Figure 4:**
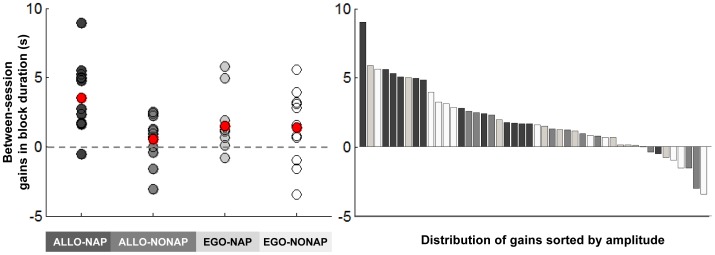
Individual offline changes in performance. Left panel: Individual between-session gains in performance for each group (s). Each point represents the difference between the average performance on the last two blocks of the representation test (RT) session and the average performance on the first two blocks of the representation retest (RR) session. Red points represent each group average in offline gain in performance. Right panel: Distribution of offline changes in performance sorted by amplitude. Note that the best quartile of the population (11 best subjects observable on the left side of the plot) is mainly composed of ALLO-NAP subjects (55%).

For completeness sake and because of the sample size in each group, individual changes in performance between sessions are presented in Figure 4. It should be noted that gains in performance in the ALLO-NAP group are highly consistent across subjects, with only one out of 13 subjects showing deterioration of performance after the nap period. On the other hand, sleep-independent gains in performance observed in the EGO groups where less consistent and robust than those observed in the ALLO-NAP group (Figure 4, left panel). Interestingly, the best quartile of the population (i.e., the 11 subjects presenting the larger offline gains in performance) was composed at 55% by ALLO-NAP subjects (6/11), 0% by ALLO-NONAP subjects (0/11), 18% by EGO-NAP subjects (2/11) and 27% by EGO-NONAP subjects (3/11, Figure 4, right panel). Altogether, results regarding individual data inspection suggest again that sleep specifically favored the emergence of gains in performance for the allocentric representation of the sequence only.

Finally, it is important to note that the significant delayed gains observed between sessions are not likely due to a continuation of the initial learning process, as stable performance was reached at the end of the representation test session: Indeed, an ANOVA testing for the saturation effect did not reveal any significant improvement over the last two blocks of practice in the representation test session in all groups (block effect, F(1,42) = 0.21, p = 0.64).

#### Accuracy during training session

An ANOVA conducted on the accuracy measure during the training session (number of errors by block; i.e. error rate), with blocks and group as within and between-subjects factors respectively, did not show significant effect of block repetition (F(13,546) = 1.05, p = 0.40). Accuracy remained stable with a low error rate (2.26±0.42 wrong key presses per block of 60 key presses) throughout training. There were also no significant group effect (F(3,42) = 1.33, p = 0.27), nor any significant block by group interaction (F(39,546) = 0.69, p = 0.92), thus indicating that subjects of the four groups had similar accuracy during training.

#### Accuracy during representation test session

In the representation test session, an ANOVA with blocks and representation as factors revealed a significant effect of block repetition (F(3,132) = 2.86, p = 0.03), whereby the level of accuracy decreased with practice. There was no significant representation effect (F(1,44) = 0.13, p = 0.71) as well as no a significant block by representation interaction (F(3,132) = 1.68, p = 0.17), suggesting that the error rate did not differ between the ALLO and EGO representations.

#### Between-session gains in performance accuracy

Between session effects were computed comparing the average error rate of the last two blocks of the representation test session against the first two blocks of the representation retest session in order to assess offline improvement in accuracy after sleep and wake periods. The ANOVA revealed no significant effect of session (F(1,42) = 1.31, p = 0.25), no significant group effect (F(3,42) = 1.06, p = 0.37), nor any group by session interaction (F(3,42) = 0.97, p = 0.41). These results indicate that the between-session changes in performance speed described above did not occur at the expense of performance accuracy.

Importantly, motor memory consolidation, as reflected by sleep-dependent performance gains in speed observed after initial motor sequence learning, has recently been questioned [38], [39]. These investigators have proposed that a gradual buildup of fatigue over the course of massed practice can negatively affect performance during late training, hence leading to an overestimation of offline performance changes between training and retest. They found that when fatigue was controlled for, the sleep enhancement effect was then substantially reduced. Yet they did not rule out a differential effect of sleep and wake on offline gains in performance.

In order to control for such confounding factors, we thus tested if (1) fatigue occurred during the course of our initial training session and if (2) fatigue induced an overestimation of the offline gains in performance observed in our experiment. Fine-grained analyses of performance speed were thus conducted by dividing the 60 trials (60 key presses) within each block into three chunks (similar to [38]), and then by computing the average time to perform the 20 key presses in each chunk in order to explore the possible emergence of within-block worsening in performance due to fatigue during the training, representation test and retest sessions.

#### Fatigue effects during training session

An ANOVA conducted on time to perform a chunk of 20 key presses with blocks (14 blocks) and chunks (3 chunks per block) as within-subject factors, as well as group (ALLO-NAP, ALLO-NONAP, EGO-NAP, EGO-NONAP) as the between-subjects factor, yielded a significant improvement in performance across blocks (F(13,546) = 33.10, p<0.001) and a significant worsening in performance across chunks within block (F(2,84) = 7.45, p = 0.001), indicating that although subjects took less time in average to produce sequences across blocks, their performance was worsening within blocks. Importantly, however, this fatigue effect did not differ between groups (F(6,84) = 0.87, p = 0.51) and no significant block by chunk interaction (F(26,1092) = 1.17, p = 0.25), nor any block by chunk by group interaction (F(78,1092) = 1.20, p = 0.11) were observed. These results suggest that fatigue effects occurring within block during initial training did not differ between groups.

#### Fatigue effects during representation test session

An ANOVA with blocks and chunks as within-subject factors and representation (ALLO vs. EGO) as the between-subjects factor revealed a significant improvement over blocks (F(3,132) = 18.94, p<0.001) but a significant worsening in performance from chunk to chunk (F(2,88) = 6.80, p = 0.001), indicating that subjects slowed down within blocks. Again the fatigue effect did not differ between representations (F(2,88) = 0.18, p = 0.82), and no significant block by chunk interaction (F(6,264) = 1.84, p = 0.09) was observed. No significant block by chunk by representation interaction (F(6,264) = 1.38, p = 0.21) was seen, suggesting also that fatigue effects did not differ across blocks and between representations during the representation test session.

#### Fatigue effects during representation retest session

The ANOVA with blocks and chunks as within-subject factors and group as the between-subjects factor yielded a significant improvement across blocks (F(3,126) = 3.04, p = 0.03), but no significant chunk effect (F(2,84) = 0.87, p = 0.42), indicating that fatigue dissipated after the 2-hour break. No significant chunk by group effect (F(6,84) = 1.13, p = 0.34), nor any significant chunk by block by group interaction (F(18,252) = 1.35, p = 0.15) were found, suggesting that the dissipation of fatigue after the 2-hour break was a non-specific effect that was not modulated by the representation, nor by the sleep/wake condition.

In conclusion, our data show a consistent worsening of performance due to repeated practice within blocks of training and representation test sessions that dissipates at retest (after a 2-hour break). This suggests that the potentially detrimental effects of fatigue on performance at the end of the representation test session might have overestimated the amount of offline gains in performance seen between test and retest sessions [38], [39]. Thus after having identified the chunks (among the three chunks of 20 key presses) that were driving the fatigue effect (see below), we re-computed offline gains in performance without those particular chunks, hence ensuring to have a measure of delayed gains less contaminated by this possible confounding factor (see below).

#### Between-session gains in performance speed after controlling for fatigue effects

Although we expected that fatigue would mainly occur on the last third chunk of each practice block (i.e., key presses 41–60), we nevertheless tested whether fatigue could also be observed on the 2nd chunk of the block (by comparing mean performance between key presses 1–20 and 21–40). A first ANOVA was then conducted comparing, for each group, the performance between the first two chunks (1–20 *vs.* 21–40) on blocks used to compute offline gains in performance (last two blocks of the representation test session and first two blocks of the representation retest session). The results did not reveal any significant chunk effect (F(1,42) = 0.81, p = 0.37), nor any chunk by group effect (F(3,42) = 1.66, p = 0.18) or chunk by block by group interaction (F(3,42) = 1.01, p = 0.39). These results show that the effect of fatigue was mainly due to a worsening in performance on the last chunk of trials within the block (i.e., key presses 41–60).

Consequently, between-session gains in performance were re-computed and analysed using the first 40 key presses thought to be less influenced by fatigue effects. The ANOVA on performance speed to perform 40 key presses with session (average performance on the last two blocks of representation test session *vs.* first two blocks of retest session) and group as within and between-subjects factors, respectively, still revealed a significant main effect of session (F(1,42) = 8.60, p = 0.005), performance improving between sessions. Yet no significant group effect (F(3,42) = 0.41, p = 0.74), nor any significant group by session interaction (F(3,42) = 1.91, p = 0.14) were now found. These results confirm those of recent behavioral studies [38], [39], which have reported that overnight gains in performance are less robust when controlled for fatigue than otherwise.

Most importantly, however, even when controlling for fatigue effects, within-group analyses still showed a significant effect of session in the ALLO-NAP group (F(1,42) = 12.22, p = 0.001), that was not observed in the ALLO-NONAP group (F(1,42) = 0.40, p = 0.52). These results show that the sleep-dependent offline gains in performance observed for the allocentric representation of the sequence was not due to a passive dissipation of fatigue, but rather to an active physiological mnemonic process that depends on sleep. By contrast, the effect of session observed in both EGO-NAP and EGO-NONAP groups were not significant anymore (EGO-NAP, F(1,42) = 2.64, p = 0.11 and EGO-NONAP, F(1,42) = 0.07, p = 0.78), hence suggesting that offline gains in these groups were probably mainly due to dissipation of fatigue effects. Even if the session by group interaction turned out to be non-significant after controlling for fatigue effects (F(3,42) = 1.91, p = 0.14), we took the liberty to perform explorative planned comparisons, corrected for multiple comparisons using the Fisher’s LSD, to compare the session effects between groups, when controlled for fatigue. As we had strong *a priori* from the first analysis (described above), we thus wanted to explore more specifically if the offline gains in performance observed in the ALLO-NAP group were strong enough to survive group comparisons after controlling for fatigue effects. These analyses revealed that the offline gains in performance (even after controlling for fatigue effects) observed in the ALLO-NAP group tended to be significantly larger than in the ALLO-NONAP and EGO-NONAP groups (ALLO-NAP *vs.* ALLO-NONAP, p = 0.06; ALLO-NAP *vs.* EGO-NONAP, p = 0.03), but did not differ from the EGO-NAP group (ALLO-NAP *vs.* EGO-NAP, p = 0.24). Also, the offline gains did not differ between the other groups (ALLO-NONAP *vs.* EGO-NAP, p = 0.48; ALLO-NONAP *vs.* EGO-NONAP, p = 0.79; EGO-NAP *vs.* EGO-NONAP, p = 0.34).

Altogether, these results suggest that fatigue indeed overestimated gains in performance in the four experimental groups as gains were reduced when controlling for this confounding factor. However, after controlling for this factor, the only persistent offline gains were observed in the ALLO-NAP group, suggesting that the emergence of delayed gains in this group was not only due to a passive dissipation of fatigue. Importantly, these offline gains in performance remain sleep-dependent for the allocentric representation of the sequence. In contrast, when controlled for fatigue, performance is only maintained (not improved anymore) for the egocentric representation of the sequence, in both NAP and NONAP groups, suggesting that the simple passage of time comprising either a sleep or wake period appears to stabilize, but not enhance, the egocentric representation. One should note, however, that the effects described above being reduced within group, the between-group differences were less robust than in the first analysis in which fatigue was not controlled for.

#### Correlation between sleep data and offline gains in performance

No significant correlations were observed between NREM or REM sleep duration or latency and offline changes in performance in the ALLO-NAP and EGO-NAP groups. However, a more detailed analysis of the NREM sleep revealed a significant correlation between spindle density (number of spindles per minute of NREM sleep reported to the total recording time) from the frontal midline derivation (Fz) and the subsequent gains in performance observed in the ALLO-NAP group (Pearson correlation test, r = 0.64, p = 0.01, Figure 3C). Notably, this correlation did hold even when offline gains in performance were controlled for fatigue effects (r = 0.62, p = 0.02). In contrast, no significant correlations were observed between offline gains in performance and spindle activity (density or size) in the EGO-NAP group on any of the derivations from which spindles were detected and extracted (Fz, Cz or Pz).

## Discussion

The aim of this study was to characterize the effect of daytime sleep (nap) on consolidation of two different representations of an explicitly learned sequence of movements. Our results show that subjects were able to transfer their sequence knowledge when the hand-keyboard coordinates were shifted, hence arguing that they acquired both a spatial (allocentric) and motor (egocentric) representation of the sequence during the training session. Our findings also demonstrate that a 90-minute nap specifically favors the consolidation of the allocentric representation of the sequence, and more specifically that NREM sleep spindles might be a marker of this process that correlates with offline gains in performance. Importantly, this pattern of results did hold irrespective of whether fatigue effects during initial practice were taken into account when computing the offline changes in performance. Yet, processing of the egocentric representation was not modulated by the sleep/wake condition.

### Transfer of Sequence Knowledge

The present study reveals that subjects did transfer their knowledge of the learned sequence, from an upright (i.e, normally oriented) hand-keyboard configuration to a new inverted position (i.e., upside-down) using the same hand. These results are consistent with those of several other studies, which have demonstrated a similar effect of sequence knowledge transfer from one hand to another [8], [10], [13], [15], [22], [47]–[50], or, as in our experiment, from one hand-keyboard configuration to another using the same hand [5], [7], [11], [12], [14], [16], [37]. The latter point is important as it indicates that the effect of transfer observed in our experiment was not confounded by the potent hemispheric dominance effect which is known to influence transfer from one hand to another [15].

Our results also show that such sequence knowledge transfer was observed for both spatial (allocentric) and motor (egocentric) representations of the sequence, as performance was faster in the representation session as compared to early training in both conditions. These findings are in line with those of numerous studies which have shown a similar dichotomy based upon a significant transfer of sequence knowledge on both representations after initial sequence learning [5], [8], [9], [11]–[13], [15], [37], [50]. This dissociation in representation is also in accord with previous integrative models, which have proposed that the acquisition of sequential behaviors resides in the dynamic interaction between different neural circuits that would encode the same motor sequence in two different coordinate systems (i.e., spatial and motor) [2]–[4]. It should be noted, however, that some other studies have reported a preferential transfer effect either on the motor [14], [16], [22], [48], [49] or spatial representation of the sequence [6], [7], [10], [47]. Yet such discrepancies could be explained by the fact that the transfer process, and the use of either representation, could depend on several factors such as the extent of practice, the task complexity, the regimen of learning [1], the level of motor skill expertise [51], and, as mentioned above, the transfer direction from one hand to another, so consequently, the hemispheric dominance [15].

Finally, our results show that the knowledge of both spatial and motor representations of the sequence is acquired in the early learning phase that is after a single training session. Although these results generally concur with Hikosaka and colleagues’ integrative model [2]–[4], they differ with respect to the dynamic process in which the two representations may develop with practice. While this model proposes that the spatial component would be preferentially created early during training and that the motor component would develop more slowly with extended practice [2]–[4], our results suggest that both spatial and motor representations exist after minimal training. Thus, we speculate that the emergence of the two sequence representations does not follow a serial model, but is rather based on parallel processes taking place during the early learning phase. Motor skill learning might then result from an integrative product of multiple neural mechanisms, each contributing to a different aspect of learning [3] that would be simultaneously elicited during the initial training session.

### Daytime Sleep Specifically Enhances Consolidation of the Allocentric Representation of the Sequence

A sleep-dependent improvement in performance speed only emerged for the allocentric representation of the sequence, and persisted even when controlling for fatigue effects. Given the sample sizes in our experiment, a close inspection of the individual data was performed and the results indicate that sleep-dependent gains in performance were consistent and robust across subjects for the allocentric representation of the sequence. In contrast, offline gains in performance for the egocentric representation of the sequence were observed irrespective of the sleep or wake condition. However, the latter gains were mainly due to a passive dissipation of fatigue at retest, as they did not hold when controlling for this confounding factor. Our results thus suggest that daytime sleep specifically enhances consolidation of the allocentric (spatial) representation of the sequence, whereas the simple passage of time comprising either a sleep or wake period appears to maintain, but not enhance, the egocentric (motor) representation as illustrated by the stabilization of performance between sessions.

Previous investigators have reported that nocturnal sleep favors the consolidation of goal-based (spatial), but not of movement-based (motor) representation of a newly learned motor sequences [9], and that it facilitates transfer of the extrinsic (spatial) [10] but not the intrinsic (motor) [37] representation of this type of motor learning. Our results are in line with those of Cohen et al. [9], and extend the findings of Witt et al. [10] and Hallgato et al. [37] who did characterize the effects of sleep and wake on transfer of sequence knowledge, but not on the consolidation *per se* of the different representations of learning. In addition, the present findings also show, for the first time, that not only a night of sleep but also a 90-minute period of daytime sleep (nap) following initial training enables the consolidation of an allocentric representation of the sequence. Finally, our results help clarify further the findings of Cohen et al. [9] on the specific role that sleep has in the consolidation of the allocentric representation of a sequence of movements. First, in our study, the protocol permitted to isolate the two representations of the sequence at the end of the initial training phase, hence ensuring that the influence of daytime sleep was properly tested on these two separate representations. Second, the use of a 90-minute nap/wake protocol in our experiment allowed us to train and retest the participants within a similar phase of the circadian cycle, hence better controlling for this confounding factor than when sessions are administered 12 hours apart [9], [10], [37]. Third, the fact that offline gains in performance were controlled for fatigue effects ensured that consolidation of the allocentric representation of the sequence seen after nap was not due to an unspecific dissipation of fatigue effect [38], [39]. In fact, even if our results revealed a progressive worsening in performance across sequences within blocks of practice during the initial training session, and a decrease in the robustness of offline gains in performance when controlled for fatigue effects, these delayed gains in performance remained significant solely in the allocentric group who experienced daytime sleep after training. One should note, however, that as gains in performance were reduced in each group when controlled for fatigue, the between-group differences were less robust than in the first analysis in which fatigue was not controlled for.

While previous findings on the effect of sleep in the expression and the consolidation of the allocentric representation of a motor sequence are consistent with our pattern of results, the role of wakefulness in processing the egocentric representation still remains unclear. Indeed, Cohen et al. [9] reported that the consolidation of this type of representation is only observed after a period of wakefulness, and not after an equivalent period of sleep, whereas our results show instead that offline gains in performance on the egocentric representation of the sequence are observed after both sleep and wakefulness when fatigue is not controlled for. When taking the latter effect into account, these offline gains were no longer significant, suggesting that they were mainly due to a passive dissipation of fatigue effect, rather than to an active offline consolidation process. In accord with Hallgato et al. [37], our result thus suggests that the consolidation of the egocentric representation depends mainly on time irrespective of the physiological state that follows the elaboration of the motor memory trace. This also indicates that the simple passage of time, including either sleep or wakefulness, does only maintain, but not improve, performance on the motor representation of the sequence. Such an interpretation is in accord with studies showing that several hours of wakefulness only help to stabilize a new motor memory trace rather than to promote the consolidation process as reflected by the emergence of offline gains in performance [25], [52], [53]. Yet it should be noted that such a stabilization effect during wakefulness has previously been found after explicit sequence learning but not after the type of implicit sequence learning used by Cohen et al. [9], for which wakefulness is rather known to promote offline gains in performance [21]. Consequently, the discrepancy between our results and those of Cohen et al. [9] regarding the role of wakefulness in the consolidation of the egocentric representation of the sequence could be explained by the nature (implicit *vs.* explicit) of the task. We speculate that in our study, the declarative knowledge for the sequence may specifically block the egocentric component of the motor skill preventing improvements from developing over wakefulness [9], [21], [54], [55].

Interestingly, the sleep-dependent gains in performance observed for the allocentric representation of the sequence were strongly correlated with the density of NREM sleep spindles during the post-training nap. Although the latter results are inconsistent with Cohen et al. [9] findings showing that the consolidation of this representation is correlated with REM sleep duration, they support those of Witt et al. [10], who reported that the expression of this representation is related to NREM stage 2 sleep duration. In fact, our results concur with an increasing number of studies that are reporting a preferential role of NREM sleep in motor sequence memory consolidation [10], [25], [26], [31]–[35]. They also agree with numerous investigations using classical motor sequence learning paradigms that are reporting a crucial role of spindles in this process during both nocturnal [32]–[35] and diurnal [26] sleep. Thus, our study confirms a correlation between sleep spindle activity and offline motor sequence memory consolidation [36] and specifies its involvement in the processing of the allocentric representation of the motor sequence.

### Possible Cerebral Mechanisms Underlying the Spatial/Motor Dichotomy

Distinct brain networks have previously been found to mediate the spatial and motor representations of a sequence [2]–[4], [6], [8], [12]. The egocentric motor representation has been shown to rely on activation of motor cortical regions, particularly the primary motor cortex [6], [8], [15] and the supplementary motor area [2], [3], [16], while the allocentric representation has been known to depend upon both parietal and prefrontal areas [2]–[4], [6]. It has been proposed that these networks would include associated striatal and cerebellar territories, and that they would be recruited following different dynamics: the neural circuit mediating the spatial representation being preferentially elicited during early training, and the network supporting the motor representation being predominantly developed later in the acquisition process [3]. Such a dissociation is in accord with results from one of our previous study, in which we reported that similar dissociable networks act in parallel for the implementation of reproducible motor behavior during initial motor sequence learning [56]. Indeed, the latter study showed that initial sequence learning was related to a progressive decrease of activity within a hippocampo-parieto-frontal network, which paralleled a cumulative increase of activity in the striatum. Thus, these previous imaging findings, together with the present behavioral results, suggest that motor sequence learning is supported by distinct networks, which are characterized by different temporal dynamics, already distinguishable during the first learning session. While the role of the striatum in motor sequence learning is well established [19], [57], there is now accumulating evidence that the hippocampus also plays an important role in this process, mainly due to its ability to associate temporally discontiguous but structured information [58], [59] and to process contingencies between perceptual features [60] (but see [61]). More particularly, this structure has been described to interact with the striatum during initial motor sequence learning [59]. Based on an analogy with spatial memory for which both allocentric and egocentric representations of space have been tested [62], it is thus tempting to speculate that the striatum and the hippocampus would, respectively, support the motor (egocentric) and spatial (allocentric) representations of the sequence during motor learning. Hence we propose that the recruitment of the hippocampus and associative areas (parieto-frontal cortices) would participate to the creation of an allocentric map of the sequence that might be processed during a subsequent sleep period leading to an enhancement in performance. Such hypothesis is in line with our previous study showing that activity in the hippocampus during initial motor sequence learning triggers sleep-dependent gains in performance [59]. In parallel, the striatum and associative cortices (mainly motor areas) would support the egocentric, motor representation of the sequence and ensure the long-term retention of that motor trace [19], [57] regardless of sleep.

Finally, with respect to the contribution of spindles in the consolidation of the allocentric representation of the sequence, it is interesting to note that these particular sleep events have been associated with hippocampal activity in animals [63] and humans [64]. Indeed, the latter authors have demonstrated that fast spindles were related to BOLD activity in the hippocampus, the medial prefrontal cortex, the pre- and post-central cortices, areas known to participate in memory consolidation [64]. In line with our hypothesis, it is then possible that spindle activity would specifically predict motor sequence memory consolidation of the allocentric, most likely hippocampal-dependent, representation of the sequence through a hippocampo-neocortical dialogue during NREM sleep (see [36] for a review). Yet these assumptions await further investigations since another study interestingly revealed correlation between spindle amplitude and changes in striatal activity after sleep-dependent motor sequence memory consolidation [35].

In conclusion, the present findings indicate that motor sequence memory consolidation is not governed by a single process, but rather involves distinct mechanisms that differently necessitate sleep or simple passage of time depending on whether the representation is allocentric or egocentric in nature. Yet the cerebral mechanisms underlying these processes, and more particularly, the respective contribution of both the striatum and the hippocampus as well as the particular role of sleep spindles, remain to be explored in order to identify the neural signatures that condition and support sleep-dependent motor sequence memory consolidation.
